# Supplementary material for: Resolving the full spectrum of human genome variation using Linked-Reads
Source: Genome Res. 2019 Apr;29(4):635–45. doi: 10.1101/gr.234443.118 (PMC6442396; doi:10.1101/gr.234443.118)
Supplement: Supplemental Material [file supp_gr.234443.118_Supplemental_longranger-2.2.zip › longranger-2.2.tar.bz2!/lr2.2-public/lib/stan/cmdstan/stan/doc/api/html/elt__divide_8hpp_source.html]

Stan: src/stan/math/matrix/elt\_divide.hpp Source File


|  |  |
| --- | --- |
|  | Stan  2.5.0  probability, sampling & optimization |


- Main Page
- Related Pages
- Namespaces
- Classes
- Files

- File List
- File Members

All Classes Namespaces Files Functions Variables Typedefs Enumerations Enumerator Friends Macros Pages

- src
- stan
- math
- matrix

elt\_divide.hpp

Go to the documentation of this file.

1 #ifndef STAN\_\_MATH\_\_MATRIX\_\_ELT\_DIVIDE\_HPP

2 #define STAN\_\_MATH\_\_MATRIX\_\_ELT\_DIVIDE\_HPP

3

4 #include <boost/math/tools/promotion.hpp>

5 #include <stan/math/matrix/Eigen.hpp>

6 #include <stan/math/error\_handling/matrix/check\_matching\_dims.hpp>

7

8 namespace stan {

9  namespace math {

10

22  template <typename T1, typename T2, int R, int C>

23  Eigen::Matrix<typename boost::math::tools::promote\_args<T1,T2>::type, R, C>

24  elt\_divide(const Eigen::Matrix<T1,R,C>& m1,

25  const Eigen::Matrix<T2,R,C>& m2) {

26  stan::math::check\_matching\_dims("elt\_divide(%1%)",m1,"m1",

27  m2,"m2",(double\*)0);

28  Eigen::Matrix<typename boost::math::tools::promote\_args<T1,T2>::type, R, C>

29  result(m1.rows(),m2.cols());

30  for (int i = 0; i < m1.size(); ++i)

31  result(i) = m1(i) / m2(i);

32  return result;

33  }

34

47  template <typename T1, typename T2, int R, int C>

48  Eigen::Matrix<typename boost::math::tools::promote\_args<T1,T2>::type, R, C>

49  elt\_divide(const Eigen::Matrix<T1,R,C>& m, T2 s){

50  return m / s;

51  }

52

65  template <typename T1, typename T2, int R, int C>

66  Eigen::Matrix<typename boost::math::tools::promote\_args<T1,T2>::type, R, C>

67  elt\_divide(T1 s,

68  const Eigen::Matrix<T2,R,C>& m) {

69  Eigen::Matrix<typename boost::math::tools::promote\_args<T1,T2>::type, R, C>

70  result(m.rows(),m.cols());

71  for (int i = 0; i < m.size(); ++i)

72  result(i) = s / m(i);

73  return result;

74  }

75

76  }

77 }

78 #endif

check\_matching\_dims.hpp

stan::math::elt\_divide

Eigen::Matrix< typename boost::math::tools::promote\_args< T1, T2 >::type, R, C > elt\_divide(const Eigen::Matrix< T1, R, C > &m1, const Eigen::Matrix< T2, R, C > &m2)

Return the elementwise division of the specified matrices.

**Definition:** elt\_divide.hpp:24

stan::math::check\_matching\_dims

bool check\_matching\_dims(const char \*function, const Eigen::Matrix< T1, R1, C1 > &y1, const char \*name1, const Eigen::Matrix< T2, R2, C2 > &y2, const char \*name2, T\_result \*result)

**Definition:** check\_matching\_dims.hpp:15

Eigen.hpp


---

[ Stan Home Page ]

*© 2011–2014,
Stan Development Team.*
